# Nationwide Genomic Surveillance of Human Respiratory Adenoviruses in 2023–2024: Evidence of Extensive Diversity and Recombination in Russia

**DOI:** 10.3390/v18010136

**Published:** 2026-01-21

**Authors:** Nikita D. Yolshin, Anna A. Ivanova, Alexander A. Perederiy, Irina V. Amosova, Tatyana A. Timoshicheva, Kirill A. Stolyarov, Daria M. Danilenko, Dmitry A. Lioznov, Andrey B. Komissarov

**Affiliations:** 1 Smorodintsev Research Institute of Influenza, 197376 Saint Petersburg, Russia; nikita.yolshin@gmail.com (N.D.Y.); anna.ivanova@influenza.spb.ru (A.A.I.); gilagalex@gmail.com (A.A.P.); irina.amosova@influenza.spb.ru (I.V.A.); tatyana.timoshicheva@influenza.spb.ru (T.A.T.); kirill@influenza.spb.ru (K.A.S.); daria.baibus@gmail.com (D.M.D.); dlioznov@yandex.ru (D.A.L.); 2 Department of Infectious Diseases and Epidemiology, Pavlov First Saint Petersburg State Medical University, 197022 Saint Petersburg, Russia

**Keywords:** human adenovirus, adenovirus C, recombinant adenoviruses, HAdV-D109, whole-genome sequencing, respiratory viruses

## Abstract

Human adenoviruses (HAdVs) are globally distributed pathogens capable of causing a wide range of clinical manifestations, particularly acute respiratory infections. However, their genomic diversity remains insufficiently characterized, with substantial geographic gaps in available sequence data, including for Russia, where only a few complete genomes have been deposited prior to this work. In this study, we analyzed more than 1200 PCR-positive respiratory specimens collected from hospitalized patients within routine surveillance projects and the Global Influenza Hospital Surveillance Network (GIHSN) across plenty of Russian regions during 2023–2024. Virus isolation followed by next-generation sequencing yielded 128 complete HAdV genomes representing species B, C, and D. The dataset included 27 B3, 9 B7, 44 B55, 12 C1, 16 C2, 4 C5, 7 C89, 5 C108, and one D109 genome, as well as three unassigned recombinant viruses with p89h5f5, p5h6f6 and p5h57f6 genomic structures (p, penton base; h, hexon; f, fiber). Phylogenetic analyses of whole genomes and capsid genes revealed extensive variability in immunogenic regions, particularly in species C, and identified clusters within B3 viruses. Notably, HAdV-D109 was identified in Russia, marking only the second reported detection of this genotype worldwide. Together, these findings substantially expand the currently available genomic landscape of HAdVs, highlighting the circulation of diverse and recombinant strains in Russia.

## 1. Introduction

Adenoviruses (AdVs) belong to the genus *Mastadenovirus* and are 90–100 nm, non-enveloped, double-stranded DNA viruses (26–45) kb [1] enclosed within an icosahedral capsid [2]. The name *adenovirus* originates from the fact that the first isolates were obtained from adenoid tissue in 1953 by Rowe et al. [3]. Since then, 116 human AdV types (Table 1) have been characterized and classified by the Human Adenovirus Working Group [4] based on viral nucleic acid content and the properties of their penton, hexon and fiber proteins; however, a substantial number of recombinant adenovirus strains remain unassigned or await formal classification. These viruses are divided into seven species, designated A–G [5], or named using a Latinized binomial format, which was implemented by the ICTV in 2023 (https://ictv.global/report/chapter/adenoviridae/adenoviridae (accessed on 18 January 2026) (Table 1).

The coxsackievirus and adenovirus receptor [6] is utilized by adenovirus types exhibiting respiratory, ocular, and gastrointestinal tropism, whereas desmoglein-2 [7] serves as a receptor mediating adenoviral infection of renal and respiratory tissues [8]. Members of the same species exhibit a high degree of similarity in nucleotide sequence and in tissue tropism [9]. Species B, C, and E primarily target the respiratory tract, causing pneumonia and acute respiratory infections [10,11]. Species B and D infect the eye [12,13], leading to keratoconjunctivitis, and species B has additionally been associated with renal and urinary tract infections. Species A, F, and G preferentially infect the gastrointestinal tract, causing gastroenteritis and diarrhea. Because case reports frequently involve immunocompromised hosts, the clinical manifestations they describe may differ from those in the general population, and discussions of tissue tropism across adenovirus subtypes can therefore appear speculative [9].

Among these, HAdVs can cause a range of diseases in humans, including self-limiting respiratory infections that may become life-threatening in immunosuppressed patients [14]. Although they are typically self-limiting, they can, for reasons that remain unclear, give rise to localized epidemics [15,16].

Until now, the study by Kurskaya et al. [17] has been the sole work to examine adenoviral genetic diversity in Russia, reporting 12 sequences of various types, exclusively from the Novosibirsk region. Their analysis revealed the circulation of genotypes C1, C2, C5, C89, and C108 among hospitalized children between 2019 and 2022. Together with the sequences presented here, those analyzed in the study by Kurskaya et al. represent the only complete genomic data from Russia deposited in NCBI that characterize the genetic diversity of adenoviruses across the country and its regions. Additionally, a case report describing adenovirus B55 detected in Moscow, including the complete genome sequence, has been published [18].

Given the limited availability of adenovirus genomic data from Russia and the considerable regional heterogeneity in viral circulation, a systematic characterization of adenoviral diversity across the country is clearly warranted. Accordingly, the aim of this study is to generate a comprehensive dataset of complete adenovirus genomes representing the breadth of viral subtypes circulating in Russia and to conduct detailed genomic analyses of these viruses. The investigation focuses on human adenoviruses detected in nasopharyngeal swab specimens, thereby primarily addressing respiratory forms of infection.

## 2. Materials and Methods

### 2.1. Sample Collection

The study utilized nasopharyngeal and oropharyngeal swab specimens obtained from both adult and pediatric patients hospitalized with acute respiratory infections (ARIs). Samples were collected within the framework of the Global Influenza Hospital Surveillance Network (GIHSN) [19] during two consecutive years, 2023 and 2024. Immediately after collection, swabs were placed into 2 mL of transport medium (UTM-RT, COPAN, Murrieta, CA, USA) and transported to the Smorodintsev Research Institute of Influenza (St. Petersburg, Russia). Upon receipt, specimens were pulse-vortexed for 15 s, aliquoted, and stored at −80 °C until further analysis. A list of participating healthcare facilities contributing respiratory samples is provided in Appendix A Table A1.

### 2.2. Genomic DNA Extraction, Virus Detection and Sequencing

Nucleic acids were extracted from 100 μL of the sample using the column-based DNA extraction kit “Genomic DNA Extraction Kit from Cells, Tissues, and Blood” (Biolabmix DU-250) (Novosibirsk, Russia), in accordance with the manufacturer’s instructions. Adenovirus was initially detected by PCR in hospital laboratories and was not retested prior to isolation procedures. Upon delivery to the Smorodintsev Research Institute of Influenza, samples with a reported Cq value below 30 were selected for isolation. Library preparation for the MGI sequencing platform was performed using the MGIEasy Fast PCR-FREE FS DNA Library Prep Set (MGITech, Shenzhen, China) according to the manufacturer’s instructions. Sequencing was carried out on an MGI DNBSEQ G-400RS (MGI Tech, Shenzhen, China) instrument with the high-throughput sequencing kit FCL SE100 (single-end read mode with a length of 100 base pairs).

### 2.3. Virus Isolation

The human lung carcinoma cell line A-549 was obtained from the collection of the Smorodintsev Research Institute of Influenza, Ministry of Health of the Russian Federation. For adenovirus isolation, A-549 cells were seeded into 96-well culture plates at a concentration of 2 × 10^5^ cells/mL and incubated at 37 °C in 5% CO_2_ for 24 h until reaching 75–90% monolayer confluence. The monolayer was then washed twice with serum-free α-MEM, after which 135 µL of maintenance medium (α-MEM supplemented with 2 mM glutamine, 100 µg/mL penicillin, and 100 µg/mL streptomycin) and 15 µL of clinical samples were added to the experimental wells. Control wells received 150 µL of maintenance medium. Plates were incubated at 37 °C in 5% CO_2_ for 72 h. The condition of the monolayer was monitored daily for cytopathic effect (CPE) characteristic of adenovirus infection using an Axiovert 40C inverted light microscope (Carl Zeiss, Jena, Germany). To increase the efficiency of adenovirus isolation, up to three consecutive passages were performed in A-549 cells. Culture supernatants from wells showing characteristic adenovirus CPE were collected and used for library preparation and sequencing.

DNA extracted from several oropharyngeal swab samples with low Cq values for adenovirus PCR was used for library preparation without prior virus isolation or amplification.

### 2.4. Genome Assembly and Consensus Correction

FastQC [20] v.0.12.1 software was used for sequence data quality assessment. Raw reads were quality-filtered and trimmed using FastP [21] v.0.23.4 was applied for quality data trimming and adapter deletion with the following parameters: removal of the first 10 bases, a minimum read length of 50 bp, a quality cutoff of Q30, and a maximum of 20% low-quality bases per read. Adapter trimming and correction of MGI read IDs were also enabled. Reads were mapped onto reference sequences using BWA-MEM2 [22] v.2.2.1 against the multi-FASTA reference panel consisting of adenovirus reference sequences obtained from NCBI and recombinant reference sequences curated by the Human Adenovirus Working Group. Alignment statistics were computed with SAMtools [23] idxstats, and the reference sequence with the highest number of mapped reads was selected as the best reference for each sample. The corresponding FASTA entry was extracted with Biopython v.1.85 and used for downstream steps. SAMtools-mpileup v.1.20 was used to produce draft consensus sequences, which were then corrected with IVAR [24] v.1.4.3. The Python Matplotlib 3.9.1 library was used to generate coverage plots.

### 2.5. Genome Annotation, Alignments and Phylogenetics

Only consensus sequences that met specific quality criteria were included in the analysis and submitted to GenBank. These criteria required 100% coverage of the hexon, penton, and fiber genes; a minimum of 95% whole-genome coverage; and a median sequencing depth greater than 10×. Sequences that did not meet these thresholds were excluded from downstream analyses.

Multiple sequence alignments of adenoviral genomes were prepared as input using MAFFT [25] v7.526 in FASTA format. Maximum likelihood phylogenetic trees were inferred using RAxML-NG [26] v1.2.2. under the General Time Reversible (GTR) model of nucleotide substitution with gamma-distributed rate heterogeneity (GTR + G) and empirical base frequency estimations (GTR + G + FO). Bootstrap analysis with 1000 replicates was performed to assess the robustness and statistical support of the inferred tree topology. Genome annotation was performed using VAPiD [27] v1.2.

### 2.6. Recombination Analysis

The open-source IDplot [28] workflow, which integrates the GARD recombination detection algorithm [29] and FastTree2 [30] into an interactive tool for recombination analysis, was used. IDplot v1.1.3 was downloaded from https://github.com/brwnj/idplot (accessed on 18 January 2026) and executed using the following command: nextflow run brwnj/idplot -latest -with-docker -alignment/path/alignment.fasta -gard -cpus 64 -gff /path/AC_000008.gff3.

Genome-wide homology was assessed using sliding window analysis (window size, 1000 bp; step size, 200 bp; substitution model, Kimura 2-parameter) implemented in the SimPlot++ tool [31].

### 2.7. Sequence Data Deposition

All nucleotide sequence data generated in this study are available in the DDBJ/EMBL/GenBank databases under accession numbers PX637903, PX121473–PX121480, PX146717–PX146760, PV692083, and PV980131–PV980201, with detailed descriptions provided in Appendix A Table A2.

### 2.8. Ethics Statement

The study was approved by the Local Ethics Committee at the Smorodintsev Research Institute of Influenza, protocol dated 24 March 2023.

## 3. Results

### 3.1. Epidemiological Characteristics of Adenovirus Circulation in the 2023/24 and 2024/25 Seasons

According to routine weekly epidemiological surveillance conducted at the Influenza Research Institute, data obtained from 37 sentinel cities showed that approximately 596,973 samples were tested during the 2023/24 and 2024/25 seasons (203,159 in 2023/24 and 393,814 in 2024/25). Of these, 6016 samples tested positive for adenovirus (2144 and 3872 for the two seasons, respectively). The proportion of adenovirus-positive cases among all tested samples was about 1% overall and within each season.

**Figure 1:**
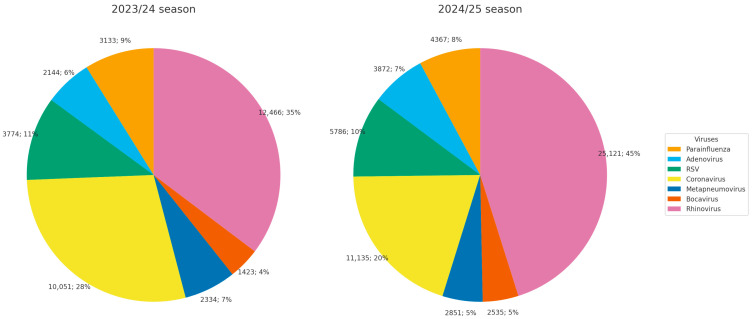
Etiological structure of diagnosed acute respiratory infections in Russia during the 2023/24 and 2024/25 seasons.

Analysis of the etiology of diagnosed viruses showed that the proportion of adenovirus among respiratory virus–positive samples (excluding influenza and SARS-CoV-2) was 6% in the 2023/24 season and 7% in the 2024/25 season (Figure 1).

The estimated annual incidence of adenoviral infection was 31.6 (30.2–32.9) and 26.7 (25.5–27.1) cases per 10,000 population in the 2023/24 and 2024/25 seasons, respectively. In absolute numbers, extrapolated to the national population, this corresponds to 461,426 (441,604–480,958) cases in 2023/24 and 389,429 (372,316–396,078) cases in 2024/25.

**Figure 2:**
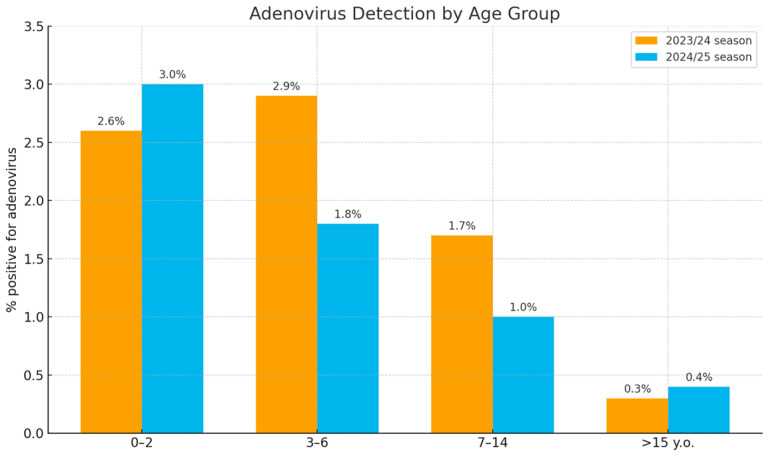
Age distribution of adenovirus-positive samples in Russia during the 2023/24 and 2024/25 seasons.

Analysis of the age distribution demonstrated that the highest detection rates (1.8% to 3.0%) occurred in children aged 0–2 and 3–6 years (Figure 2). In the 7–14-year age group, detection rates were lower (1.0% to 1.7%). The lowest rates (0.3% to 0.4%) were observed in the oldest age group (15 years and above).

### 3.2. Overview of Sample Cohort and Genomic Dataset

In total, more than 1200 human adenovirus–positive samples, representing a subset of adenovirus-positive cases identified through national epidemiological surveillance, were received from participating hospitals in multiple shipment batches and processed during the study period. Over 200 viruses were successfully isolated and sequenced; however, complete genome assemblies were obtained only for a subset of these samples. Ultimately, 128 samples yielded high-quality genome sequences and were included in the present analysis.

**Figure 3:**
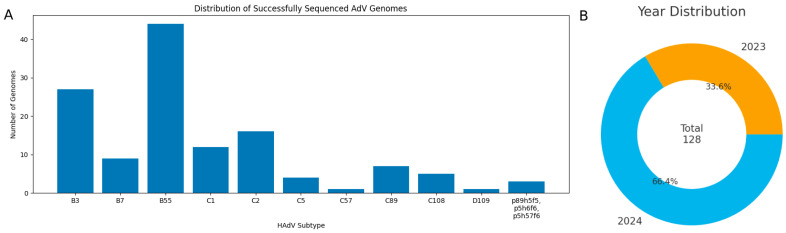
(**A**) Distribution of successfully sequenced HAdV genomes. (**B**) Distribution of genome sequences by year of sample collection.

In this study, we present 128 complete HAdV genomes from 16 Russian regions along with their genetic analysis. Among them are 27 B3 genomes, 9 B7, 44 B55, 12 C1, 16 C2, 4 C5, 7 C89, 5 C108, and one D109. Three more samples were found to contain recombinant viruses p89h5f5, p5h6f6 and p5h57f6, where p/h/f denote the penton base, hexon, and fiber genes, respectively. The distribution of successfully sequenced HAdV genomes, stratified by type and year of sample collection, is presented in Figure 3.

The successfully sequenced samples originated from 16 regions: Astrakhan Region, Khabarovsk Region, Komi Republic, Krasnodar Region, Lipetsk Region, Novosibirsk Region, Perm Region, Pskov Region, Republic of Bashkortostan, Republic of Buryatia, Republic of Tatarstan, Rostov Region, Saint Petersburg, Tambov Region, Vologda Region, and Yamalo-Nenets Autonomous Region.

**Figure 4:**
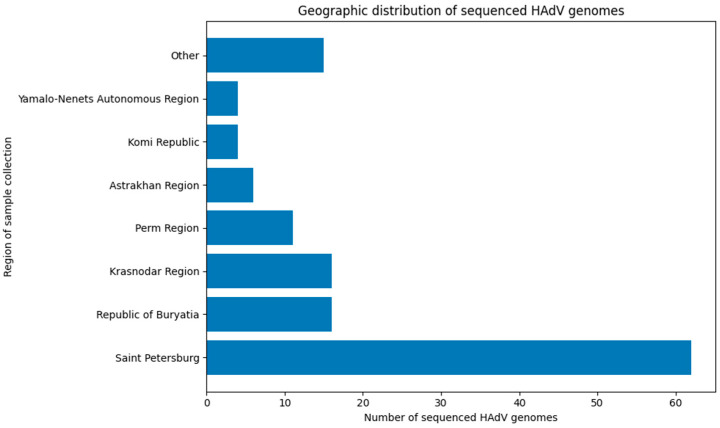
Distribution of successfully sequenced adenovirus samples by region (regions with 1 or 2 samples combined as ‘Other’).

However, sampling was geographically skewed, with the majority of samples originating from Saint Petersburg. Consequently, the dataset cannot be considered fully representative for assessing adenoviral genetic diversity across the entire country or for drawing definitive conclusions about the relative proportions of circulating viruses. Nevertheless, the data provide valuable insights into a problem that has, until now, remained largely unexplored. The geographic distribution of genetically characterized sequences is summarized in Figure 4.

### 3.3. Virus Isolation and Virological Characteristics

**Figure 5:**
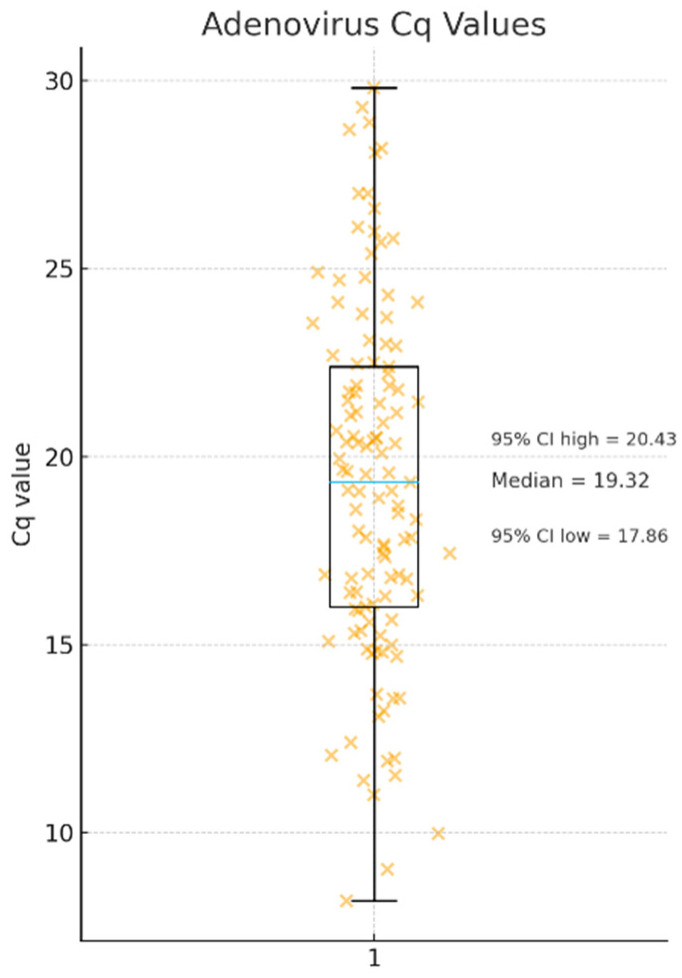
Distribution of adenovirus Cq values obtained from clinical respiratory specimens that yielded successful whole-genome assemblies. Orange “×” symbols represent Cq values for single clinical specimens, while the blue horizontal line within the box indicates the median Cq value (19.3) with a 95% confidence interval ranging from 17.8 to 20.4, *n* = 128.

Only samples with Cq values below 30 were used for isolation, and the median Cq among those that yielded successful whole-genome assemblies was 19.3 (Figure 5).

**Figure 6:**
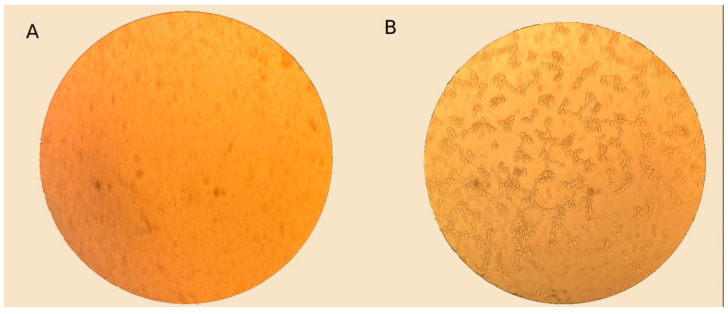
Light microscopy images of A-549 cells: infected with clinical material and control, magnification ×400. (**A**) Control A-549 cell culture. (**B**) Cell culture A-549, 72 h after infection with clinical material. Cytopathic effect (CPE) characteristic of adenovirus is observed.

To increase the efficiency of adenovirus isolation, up to three successive passages were performed in A-549 cell culture. The virus-containing culture fluid from the wells that exhibited a cytopathic effect characteristic of an adenovirus was subsequently used for sequencing (Figure 6).

### 3.4. Sequencing

Only genomes that fulfilled predefined quality thresholds were retained for analysis and deposition in GenBank. Specifically, sequences were included only when the hexon, penton, and fiber genes were fully covered, whole-genome coverage exceeded 95%, and the median sequencing depth was greater than 10×. Genomes not meeting these criteria were excluded from further analysis.

The median sequencing coverage for the assembled adenovirus consensus genomes varied widely across samples, reflecting substantial differences in viral load, sequencing depth, and library quality. Coverage values ranged from 11× to 4865× (several high-quality samples) with the median coverage approximately 170×. Mapped read counts ranged from below 4000 to over 900,000 reads per sample, also reflecting substantial variability in sequencing depth.

Samples #1092, #1100 and #1124 (PV980131–PV980133) with low Cq values were additionally subjected to direct sequencing to test the hypothesis that successful genome recovery is possible without prior isolation. DNA extracted from several oropharyngeal swab samples was used for library preparation without virus isolation or amplification, and genomes with at least 10× coverage were successfully assembled, using the same estimated number of sequencing reads as for isolate-derived genomes (approximately one million reads per sample).

### 3.5. Phylogenetic and Genomic Analyses

The complete genomes of 128 HAdV isolates were successfully sequenced and deposited in the NCBI GenBank (accession numbers indicated in Appendix A Table A2). We then conducted phylogenetic analyses of these isolates together with representative HAdV sequences available in the GenBank database.

#### 3.5.1. Adenovirus B

**Figure 7:**
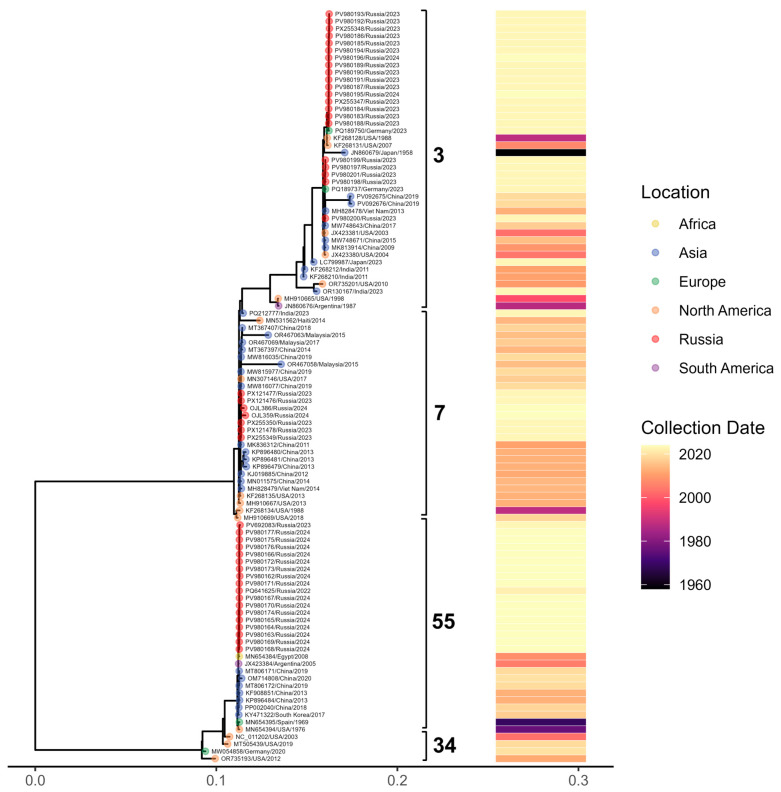
Maximum-likelihood phylogenetic tree of Adenovirus B whole-genome sequences representing four major subtypes. Tips are colored by geographic region (with samples from Russia shown in red), brackets denote major clades, and the accompanying heatmap indicates the year of sample collection.

To contextualize the sequences generated in our laboratory within the broader evolutionary landscape of Adenovirus B, we constructed a maximum-likelihood phylogeny that includes both our newly obtained isolates and representative sequences retrieved from GenBank (Figure 7). The resulting tree resolves four major clades, each characterized by distinct temporal and geographic patterns. Most of our newly generated sequences cluster within three of these clades (27 B3 genomes, 9 B7, 44 B55), exhibiting minimal divergence and indicating close genetic relatedness.

HAdV-B3 sequences produced in this study display multiple amino acid substitutions and segregate into two well-supported clusters according to their variation profiles. Additionally, a single-amino-acid S insertion in position 21 was identified in the B3 penton base for PV980200.1. By comparison, the immunogenic capsid protein sequences of the nine B7 and 44 B55 genomes generated here showed no detectable variation.

#### 3.5.2. Adenovirus C

**Figure 8:**
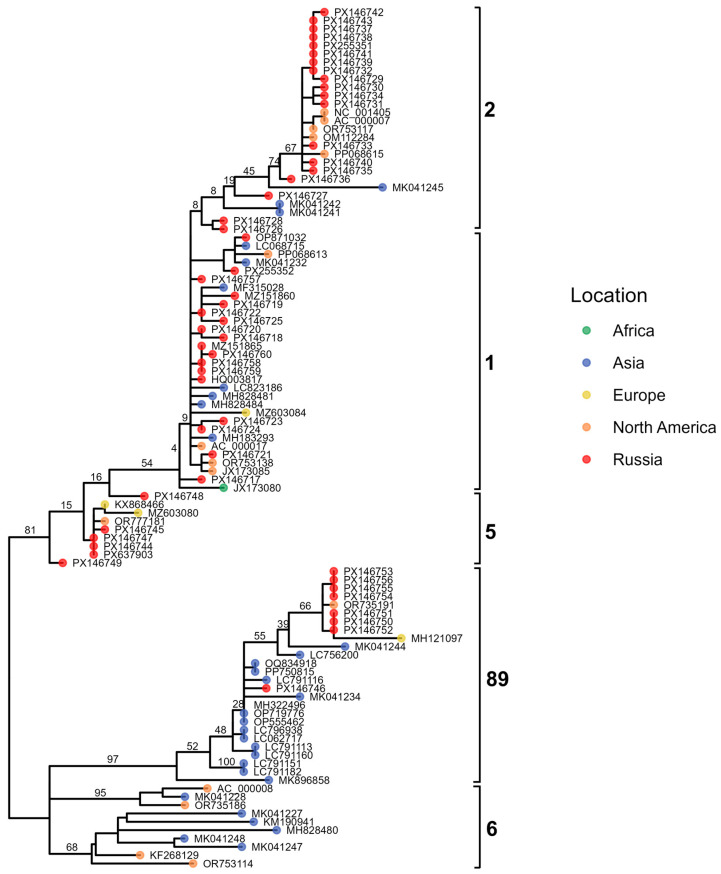
Maximum-likelihood phylogenetic tree of Adenovirus C penton gene sequences. Tips are colored by geographic region, with Russian isolates shown in red. Major genotypes are indicated by brackets.

**Figure 9:**
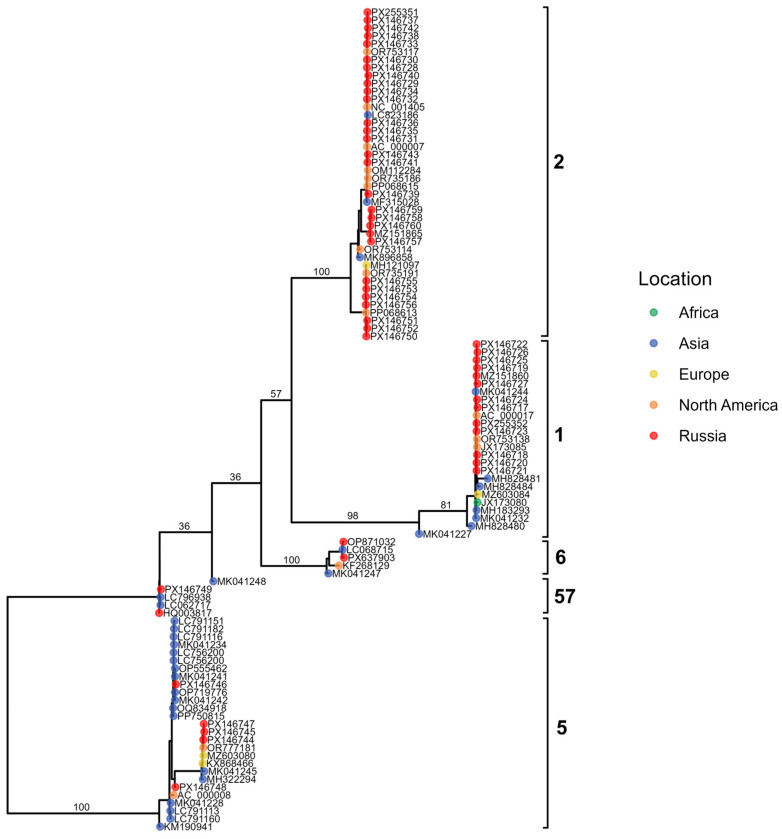
Maximum-likelihood phylogenetic tree of Adenovirus C hexon gene sequences. Tips are colored by geographic region, with Russian isolates shown in red; brackets highlight major genotypes.

**Figure 10:**
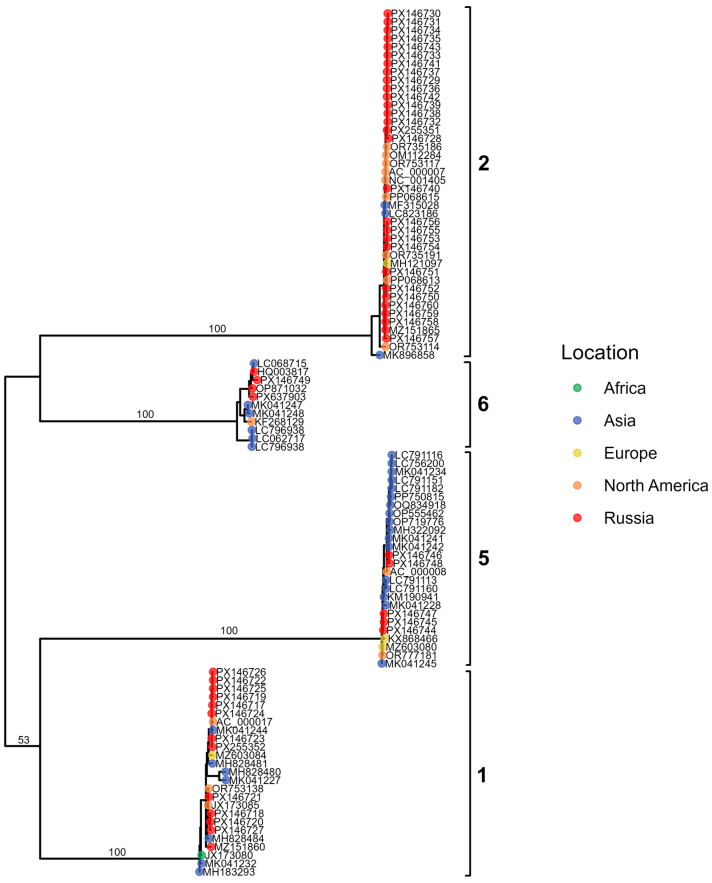
Maximum-likelihood phylogenetic tree of Adenovirus C fiber gene sequences. Tips are colored by geographic region, with Russian isolates shown in red. Major genotypes are indicated by brackets.

To characterize the genetic structure of our Adenovirus C isolates and assess the presence of potential recombinants, we constructed maximum-likelihood phylogenies for the three major capsid genes: penton base (Figure 8), hexon (Figure 9), and fiber (Figure 10). These gene-specific trees allowed us to compare our sequences with representative global strains and to evaluate whether individual genomic regions grouped consistently within established HAdV-C genotypes.

**Figure 11:**
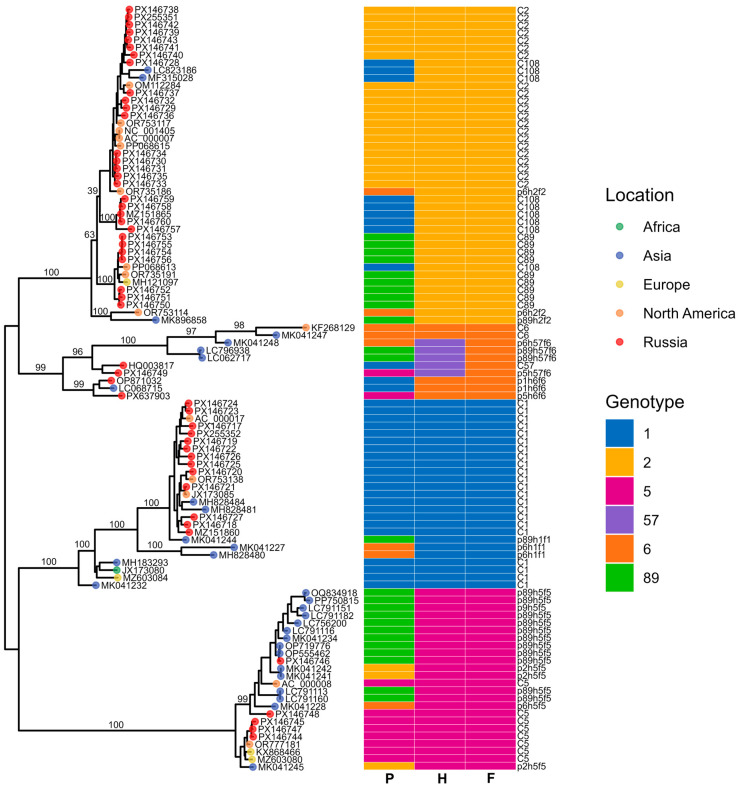
Maximum-likelihood phylogenetic tree of Adenovirus C whole-genome sequences. Tips are colored by geographic region, with Russian samples shown in red. The heatmap indicates genotype assignment across the three genomic regions (P, H, and F). Brackets denote major clades.

In addition to the three gene-specific phylogenies, we also reconstructed a whole-genome maximum-likelihood tree to provide an integrated evolutionary framework for the Adenovirus C sequences (Figure 11). The whole-genome phylogeny was complemented by a visualization of genome structure, in which the capsid gene assignments inferred from the penton, hexon, and fiber trees were mapped onto each sequence. This approach enabled us to compare overall genomic relatedness with capsid–gene-specific clustering patterns and to detect signatures of recombination across the genome.

The resulting phylogenies revealed clear clustering into the known genotypes (12 C1, 16 C2, 4 C5, 7 C89, 5 C108), enabling us to assign capsid gene types for each isolate. Notably, three recombinant viruses lacking assignment to any established genotype—p89h5f5, p5h6f6 and p5h57f6—were detected, each defined by unique combinations of capsid gene types.

Within the C5 genotype, the four genomes segregated into two distinct groups according to their amino-acid substitution profiles. Additional substitutions were observed in the fiber and penton proteins of the C1, C2, and C108 genomes, whereas the hexon proteins of these types showed no detectable variation. Notably, the C2 penton base exhibited both a single amino-acid deletion G10del (PX146727.1).

**Figure 12:**
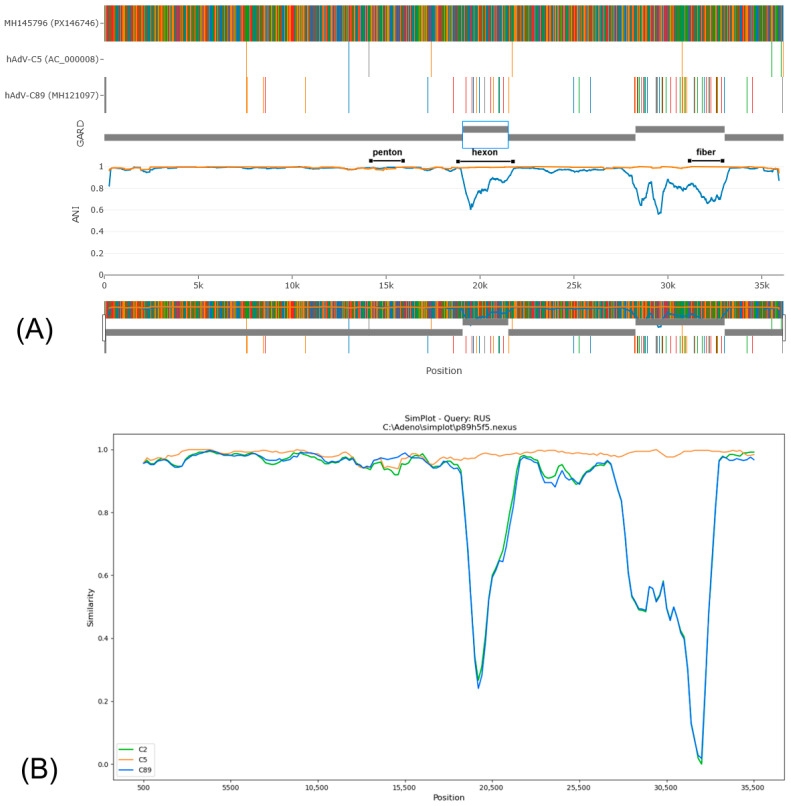
(**A**) IDPlot analysis of HAdV-C/Russia/BU-RII-MH145796V/2023 in comparison with HAdV-C5 (orange line) and HAdV-C89 (blue line). (**B**) SimPlot analysis of HAdV-C/Russia/BU-RII-MH145796V/2023 in comparison with HAdV-C5 (orange line), HAdV-C2 (green line), and HAdV-C89 (blue line).

Based on phylogenetic analysis of the penton gene, HAdV-C/Russia/BU-RII-MH145796V/2023 (GenBank Acc. No. PX146746) was assigned to genotype 89. In contrast, phylogenetic trees inferred from the hexon and fiber genes show that this virus clusters with strains belonging to genotype 5. IDPlot analysis (see Figure 12) revealed extended genomic regions that distinguish HAdV-C/Russia/BU-RII-MH145796V/2023 from typical representatives of genotype 89. In particular, the hexon and fiber regions show the highest similarity to HAdV-C5. Notably, the putative recombinant segment at the fiber locus is substantially broader, spanning nt 28,275–33,020, and encompasses not only the fiber gene but also the E3 region, which encodes E3 12.5K, E3 CR1-α, E3 gp19K, 10.5 kDa, E3 RID-α, E3 RID-β, E3 14.7K, and the U exon. The IDPlot findings were corroborated by SimPlot analysis (Figure 12B).

**Figure 13:**
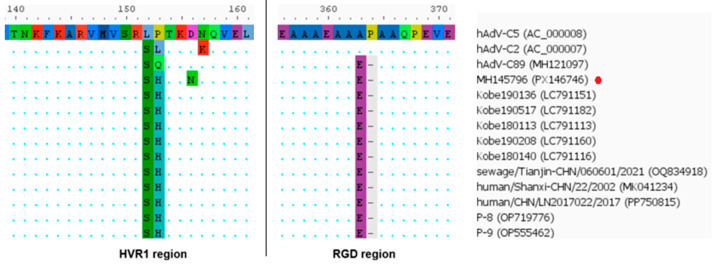
Amino acid substitutions in the penton protein of p89h5f5 strains, including HAdV-C/Russia/BU-RII-MH145796V/2023 (indicated by a red dot).

The penton sequences of genotypes 89 and 5 differ only slightly, and these differences are not attributable to recombination. Accordingly, in Figure 12, the penton similarity plots for genotype 89, genotype 5, and HAdV-C/Russia/BU-RII-MH145796V/2023 almost completely overlap. To confirm that the penton of HAdV-C/Russia/BU-RII-MH145796V/2023 belongs to genotype 89, we analyzed the amino acid sequence to identify a characteristic substitution profile. Amino acid sequence analysis of the penton revealed a substitution in HVR1 at position 153: P153Q in the genotype 89 reference strain and P153H in HAdV-C/Russia/BU-RII-MH145796V/2023. Of note, the HAdV-C/Russia/BU-RII-MH145796V/2023 strain also carries an additional HVR1 substitution, D156N. In the penton loop, the RGD region contains the A363E substitution and a deletion at position 364 (P364del), both of which are characteristic of genotype 89 (Figure 13).

Whole-genome phylogenetic analysis identified adenovirus genomes from China and Japan that are closely related to HAdV-C/Russia/BU-RII-MH145796V/2023. Separate phylogenetic analyses of the capsid genes confirmed that these viruses belong to the p89h5f5 recombinant. Key metadata for these viruses are summarized in Table 2.

Phylogenetic analysis of complete genomes indicates that these viruses form a single cluster (with the exception of Kobe180113 and Kobe190208), which likely originated from genotype 5 adenoviruses. Exceptions are represented by two genomes from Kobe Prefecture, Japan—Kobe180113 (LC791113) and Kobe190208 (LC791160) [35]. These viruses conform to the p89h5f5 formula based on the penton, hexon, and fiber genes; however, analysis of genomic regions outside these loci indicates the presence of additional recombination events. Specifically, increased similarity to genotype 1 sequences was observed in the polymerase and E4 regions (see Supplementary Figures S1 and S2).

#### 3.5.3. Adenovirus D109

To our knowledge, this represents the second reported detection of human adenovirus type D109 (HAdV-D109) worldwide. In the previously described case involving a one-year-old infant, clinical manifestations were typical of an acute respiratory infection, including fever, chills, cough, diarrhea, nasal congestion, and sore throat. In contrast, the present case involved a 43-year-old woman who required hospitalization and exhibited a severe clinical presentation characterized by fever, general malaise, sore throat, prominent ocular involvement (eye pain and photophobia), multiple painful erosions of the oral mucosa, and rapidly progressive diffuse cutaneous lesions with blistering, indicating a severe disease course. Rhinovirus was also detected concomitantly with adenovirus.

**Figure 14:**
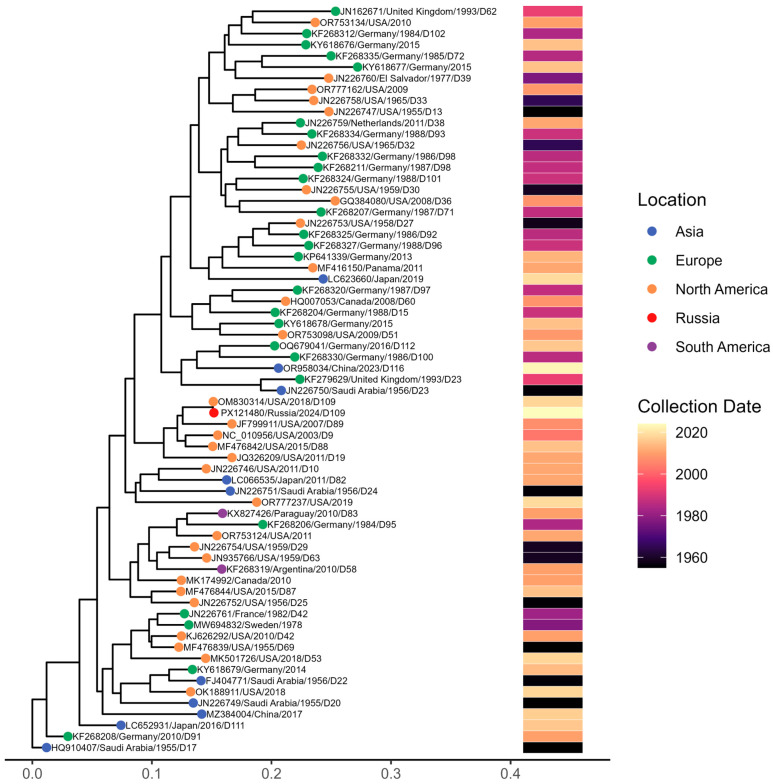
Maximum-likelihood phylogenetic tree of Adenovirus D whole-genome sequences. Tips are colored by geographic region, and the accompanying heatmap shows collection dates from the 1950s to 2024.

To contextualize the single Adenovirus D genome (D-109) identified in our study, we reconstructed a whole-genome maximum-likelihood phylogeny that includes representative HAdV-D sequences spanning multiple decades and geographic regions (Figure 14). The resulting tree shows extensive global heterogeneity within the HAdV-D group, where our D-109 sample clusters within one of the major HAdV-D lineages, closely grouping with contemporary isolates from North America and Europe.

Comparative genomic analysis revealed a very high level of similarity to the previously reported isolate 592275 (GenBank accession no. OM830314.1), with 99.94% nucleotide sequence identity, indicating minimal genomic divergence; consistent with this, both isolates clustered together in the phylogenetic tree.

## 4. Discussion

Adenoviruses undergo recombination and mutation, generating new variants with altered pathogenicity or transmissibility [38]. Genomic data provide valuable insights into these evolutionary processes. Monitoring adenoviral genetic diversity is essential for identifying emerging strains and adapting diagnostic assays, and it is also important given that adenoviruses are widely used in biotechnology as vectors for gene delivery, vaccine development, oncolytic cancer therapy, etc.

Information on genetic diversity remains highly uneven across pathogens. While millions of SARS-CoV-2 sequences are available, fewer than 2500 complete human adenovirus genomes have been deposited in GenBank, with only a few dozen represented for certain subtypes. Geographic representation is equally limited: for Russia, fewer than ten complete adenovirus genomes were available prior to our contribution. In fact, the global dataset on adenoviral genetic diversity remains insufficient overall, not merely at the regional level. The sequences generated in this study therefore provide a meaningful contribution to the global knowledge base on adenoviruses. For example, only two sequences of adenovirus D109 are currently available in NCBI: the reference genome and the sequence reported here.

The present study reports whole-genome sequences of HAdV isolates obtained from hospitalized patients with respiratory disease across different regions of Russia. The analysis revealed insights into the genetic diversity of circulating adenoviruses and identified several novel recombinant strains. The findings predominantly reflect adenoviruses causing respiratory disease, given that the analysis was limited to oropharyngeal swab specimens.

Over the past decade, advances in sequencing technologies that enable analysis of the entire viral genome have led to the identification of numerous recombinant types. Nevertheless, although the number of recognized HAdV types has increased rapidly, information regarding the clinical significance of these recombinant forms remains limited [39]. Genomic regions of HAdV that are most commonly involved in homologous recombination include the penton base, hexon, and fiber coding regions, which also represent the loci most frequently affected by mutations [40]. Recombinant adenoviruses do not necessarily possess entirely new genome sequences; rather, they may represent novel combinations of existing coding regions. For example, C89 and C57 contain genuinely new capsid protein sequences (p89 and h57, respectively), whereas C104 and C108 are not defined by unique sequence innovations but instead represent new genomic configurations—p1h1f2 for C104 and p1h2f2 for C108.

In our study, excluding the well-known recombinants B55, C89, C108, and D109, we identified three additional viruses that are likely unassigned recombinants. The first, PX146746.1, exhibits a p89h5f5 genome structure. In GenBank, 11 genomes share the same p89h5f5 profile, all of which are broadly classified as HAdV-C. The hAdV-C/Russia/BU-RII-MH145796V/2023 p89h5f5 recombinant virus identified in the Republic of Buryatia in 2023 shows high similarity to viruses detected in multiple provinces of China during 2002–2021 [32,33,36,37], as well as to viruses from Japan (Aichi and Kobe Prefectures) collected in 2017–2019 [34,35]. Notably, genotype 89 was first identified in fecal samples, and a substantial proportion of specimens containing the p89h5f5 recombinant form also originate from the gastrointestinal tract (Table 2). This observation suggests that these viruses may exhibit dual tropism for both the respiratory and gastrointestinal tracts. Of additional concern, the p89h5f5 group includes strain human/CHN/LN2017022/2017, which was recovered from a stool specimen collected from a patient with acute flaccid paralysis [33]. There is strong evidence that p89h5f5 recombinant viruses form a distinct, monophyletic clade in the whole-genome phylogenetic tree, with two exceptions: Kobe180113 and Kobe190208 strains. These two genomes fall outside the main clade, plausibly due to additional recombination events affecting the polymerase and E4 regions. The oldest representative of this lineage dates to 2002, suggesting that the p89h5f5 recombinant represents an East Asia–origin lineage that has been circulating cryptically for more than 24 years, causing sporadic infections in China and Japan over time. More broadly, recombination events outside the canonical “penton–hexon–fiber” gene set may substantially contribute to adenovirus genetic diversity, but this is likely underestimated under the current PHF-focused nomenclature.

A second virus without an established genotype, PX637903, displays a p5h6f6 structure. In GenBank, only two sequences (MH121112 and MH121113) show >99% similarity to this genome. These sequences were generated in the study by Dhingra et al. [41], which investigated adenovirus evolution and formally designated the C89 recombinant.

The third sequence generated in this study (PX146749.1) exhibited a p5h57f6 genomic structure. This genome shares very high similarity (>99%) with the Adenovirus Working Group reference strain HQ003817, which is classified as HAdV-C57 [42] (p1h57f6). However, in our phylogenetic analyses, HQ003817 consistently grouped as p1h57f6, whereas PX146749.1 clustered as p5h57f6, indicating that despite their overall genomic similarity, the penton gene of PX146749.1 corresponds to a different lineage.

It has long been assumed that, unlike HAdV-D, the evolution of HAdV-C is not characterized by recombination affecting the penton, hexon, and fiber genes. Accordingly, the emergence of genotype 89 was proposed to have resulted primarily from mutational changes in the HAdV-C2 penton and recombination in the E4 region [41]. However, the identification of HAdV-57 (p1h57f6) [42], HAdV-104 (p1h1f2) [43], HAdV-108 (p1h2f2) [44], and p89h5f5 suggests that the contribution of recombination to HAdV-C evolution warrants reassessment. It is clear that the current adenovirus classification system, which is based on the penton, hexon, and fiber genes, fails to capture substantial diversity generated by recombination in non-capsid genomic regions.

In addition to recombination events, we also identified a series of mutations in the immunogenic capsid proteins. The 27 B3 sequences generated in this study differed by multiple amino-acid substitutions and could be grouped into two distinct clusters based on these variation patterns. In contrast, the immunogenic capsid protein sequences of the 9 B7 and 44 B55 genomes obtained in our study showed no detectable variation. Among penton base, hexon, and fiber, the greatest diversity was observed in the fiber protein.

The greatest type-level diversity was observed among adenoviruses of species C (12 C1, 16 C2, 4 C5, 7 C89, and 5 C108). The four C5 genomes clustered into two groups based on their amino-acid substitution patterns. Additional amino-acid substitutions were identified in the fiber and penton proteins of C1, C2, and C108 genomes, whereas no substitutions were detected in the hexon protein for these types.

Several indels in the immunogenic capsid proteins were localized to the penton base, with no insertions or deletions detected in either the hexon or fiber genes. Specifically, a single–amino-acid insertion was observed in the B3 penton base in one sample, and a single–amino-acid deletion was identified in the C1 penton base.

Adenovirus D109 was first described in January 2018 [45] during surveillance at the California/Mexico border and was associated with acute respiratory disease. To the best of our knowledge, this subtype (p22h19f9) has not been identified elsewhere since its initial description. Comparative genomic analysis demonstrated 99.94% nucleotide sequence identity with the previously reported isolate 592275 (GenBank accession no. OM830314.1), indicating minimal genomic divergence. The limited clinical information provided by this case may nevertheless be valuable for future case identification and for improving the understanding of the potential pathogenic spectrum of HAdV-D109.

Although all of our results were obtained from sequencing adenoviral isolates, our preliminary experiments demonstrate that adenoviral samples with low Cq values can be successfully subjected to direct sequencing. DNA extracted directly from oropharyngeal swab samples was used for library preparation without prior virus isolation or amplification. Under these conditions, genomes achieving at least 10× coverage were successfully assembled against the reference. However, our experiment with direct sequencing was limited in scope, and additional work is required to more thoroughly evaluate sample quality parameters that enable efficient genome recovery without enrichment steps. Further studies are also needed to determine whether direct sequencing is a cost-effective and practical approach for routine adenovirus genomic surveillance. At the present stage, we point to the possibility of direct sequencing of adenoviruses from clinical swab samples using the same estimated number of sequencing reads as for isolate-derived genomes, with a relatively low read allocation per sample (approximately one million reads per sample).

Subtyping of human adenoviruses by PCR is currently restricted mainly to reference laboratories, research studies, or outbreak investigations. Given the large and continuously expanding number of recognized HAdV types, NGS represents a more comprehensive approach for adenovirus subtyping in clinical settings, particularly if whole-genome sequencing can be performed directly from clinical material without prior virus isolation. With respect to the development of other molecular techniques for HAdV subtyping, detailed knowledge of mutations and recombination events in immunogenic capsid proteins is highly relevant for diagnostic assay design, as sequence variation may compromise the performance of PCR primer/probe sets or antigen-based detection methods.

### Limitations of the Study

This study was conducted using samples collected from different regions of Russia; however, their distribution was uneven and therefore may not fully represent the nationwide epidemiology of adenoviruses. In addition, the absence of clinical data limits the ability to assess potential associations between viral genotypes and disease severity or clinical outcomes. Another limitation is that only respiratory specimens (swabs) were analyzed, which restricts conclusions about adenovirus diversity in other types of infections.

## Figures and Tables

**Table 1:** Distribution of Human Adenovirus Types Across Species With Corresponding ICTV Species Names.

Species	ICTV Name	Types	Total
A	*Mastadenovirus adami*	12, 18, 31, 61	4
B	*Mastadenovirus blackbeardi*	3, 7, 11, 14, 16, 21, 34, 35, 50, 55, 66, 68, 76, 77, 78, 79, 106, 114	18
C	*Mastadenovirus aesari*	1, 2, 5, 6, 57, 89, 104, 108	8
D	*Mastadenovirus dominans*	8–10, 13, 15, 17, 19, 20, 22–30, 32, 33, 36–39, 42–49, 51, 53, 54, 56, 58–60, 62–65, 67, 69–75, 80–88, 90–103, 105, 107, 109–113, 115, 116	82
E	*Mastadenovirus exoticum*	4	1
F	*Mastadenovirus faecale*	40, 41	2
G	*Mastadenovirus russelli*	52	1

**Table 2:** Recombinant p89h5f5 adenoviruses available in GenBank.

GenBank Acc. No.	Isolate Name	Country, Province	Collection Date	Specimen Type	Ref.
MK041234	human/Shanxi-CHN/22/2002	China, Shanxi	19 January 2002	Fecal	[32]
PP750815	human/CHN/LN2017022/2017	China, Liaoning	2017	Fecal	[33]
LC756200	Aichi39_2017	Japan, Aichi	2017	Fecal	[34]
LC791113	Kobe180113	Japan, Kobe	2018	Respiratory	[35]
LC791116	Kobe180140	Japan, Kobe	2018	Respiratory	[35]
LC791151	Kobe190136	Japan, Kobe	2019	Respiratory	[35]
LC791160	Kobe190208	Japan, Kobe	2019	Respiratory	[35]
LC791182	Kobe190517	Japan, Kobe	2019	Respiratory	[35]
OP555462	P-9	China, Yunnan	23 April 2021	Fecal	[36]
OP719776	P-8	China, Yunnan	3 May 2021	Fecal	[36]
OQ834918	sewage/Tianjin-CHN/060601/2021	China, Tianjin	June 2021	Sewage	[37]
PX146746	HAdV-C/Russia/BU-RII-MH145796V/2023	Russia, Republic of Buryatia	26 September 2023	Respiratory	this paper

## Data Availability

All nucleotide sequence data generated in this study are available in the DDBJ/EMBL/GenBank databases under accession numbers PX637903, PX121473–PX121480, PX146717–PX146760, PV692083, and PV980131–PV980201, with detailed descriptions provided in Table A2, Appendix A.

## Supplementary Materials

The following supporting information can be downloaded at: https://www.mdpi.com/article/10.3390/v18010136/s1, Figure S1: SimPlot analysis of Kobe180113 (LC791113) and Kobe190208 (LC791160) strains (brown line) in comparison with HAdV-C5 and p89h5f5 (pink line). Figure S2: Maximum-likelihood phylogenetic tree of Adenovirus C E4 region sequences. Tips are colored by geographic region, with Russian samples shown in red. Kobe180113 (LC791113) and Kobe190208 (LC791160) strains are indicated with red arrows. The heatmap indicates genotype assignment across the three genomic regions (P, H, and F).

## Abbreviations

The following abbreviations are used in this manuscript:

HAdV	Human adenovirus
ARI	Acute respiratory infection
CPE	Cytopathic effect
Cq	Quantification cycle
GenBank	International nucleotide sequence database
GIHSN	Global Influenza Hospital Surveillance Network
GTR	General Time Reversible
ML	Maximum likelihood
NCBI	National Center for Biotechnology Information
NGS	Next-generation sequencing
PCR	Polymerase chain reaction
UTM	Universal Transport Medium

## Appendix A

**Table A1:** List of Healthcare Facilities Providing Respiratory Disease Patient Samples.

Institution Name	Region	City
State Budgetary Healthcare Institution “Republican Clinical Infectious Diseases Hospital”	Republic of Buryatia	Ulan-Ude
St. Petersburg State Budgetary Healthcare Institution “Children’s City Polyclinic No. 35”	Saint Petersburg	Saint Petersburg
Astrakhan Regional Clinical Infectious Diseases Hospital named after A.M. Nichogi	Astrakhan Region	Astrakhan
Perm Regional Clinical Infectious Diseases Hospital	Perm Territory	Perm
Perm State Budgetary Healthcare Institution “City Children’s Polyclinic No. 1”	Perm Territory	Perm
Lipetsk Regional Clinical Infectious Diseases Hospital	Lipetsk Region	Lipetsk
Sheksninskaya Central District Hospital	Vologda Region	Sheksna
Perm Regional Infectious Diseases Hospital	Perm Territory	Perm
Chaykovsky Central City Hospital	Perm Territory	Chaykovsky
Specialized Clinical Children’s Infectious Diseases Hospital	Krasnodar Territory	Krasnodar
St. Petersburg Clinical Infectious Diseases Hospital named after Botkin	Saint Petersburg	Saint Petersburg
Children’s City Polyclinic No. 4 of Krasnodar	Krasnodar Territory	Krasnodar
Children’s City Polyclinic No. 5 of Krasnodar	Krasnodar Territory	Krasnodar
Infectious Diseases Hospital No. 3 of Novorossiysk	Krasnodar Territory	Novorossiysk
Infectious Diseases Hospital No. 2	Krasnodar Territory	Sochi
Krymsk Central District Hospital	Krasnodar Territory	Krymsk
Specialized Clinical Children’s Infectious Diseases Hospital	Krasnodar Territory	Krasnodar
Noyabrsk Central City Hospital	Yamalo-Nenets Autonomous Okrug	Noyabrsk
St. Petersburg Children Infectious Diseases Hospital No. 3	Saint Petersburg	Saint Petersburg
Children’s Polyclinic of the Railway District, Rostov-on-Don	Rostov Region	Rostov-on-Don
Children’s City Hospital (Komsomolsk-on-Amur)	Khabarovsk Territory	Komsomolsk-on-Amur
Republican Children’s Clinical Hospital	Republic of Bashkortostan	Ufa
Smorodintsev Research Institute of Influenza	Saint Petersburg	Saint Petersburg
St. Olga Children’s City Hospital	Saint Petersburg	Saint Petersburg
Republican Clinical Infectious Diseases Hospital	Republic of Tatarstan	Kazan
Children’s Polyclinic No. 2 of Krasnodar (Vostochnokruglikovskaya)	Krasnodar Territory	Krasnodar
Republican Infectious Diseases Hospital	Komi Republic	Syktyvkar
Novosibirsk Children’s City Clinical Hospital No. 6	Novosibirsk Region	Novosibirsk
Infectious Diseases Hospital No. 3 of Novorossiysk	Krasnodar Territory	Novorossiysk
Children’s Infectious Diseases Department	Pskov Region	Pskov
Rasskazovo Central District Hospital	Tambov Region	Rasskazovo

**Table A2:** Sample Metadata and GenBank Accession Numbers for Sequenced Adenovirus Genomes.

Region	City	Subtype	Lab #	NCBI #
Republic of Buryatia	Ulan-Ude	B7	MH145774	PX121473.1
Republic of Buryatia	Ulan-Ude	B7	MH145795	PX121474.1
Republic of Buryatia	Ulan-Ude	p89h5f5	MH145796	PX146746.1
Saint Petersburg	Saint Petersburg	B3	MH147312	PV980183.1
Astrakhan Oblast	Astrakhan	B3	MH147400	PV980184.1
Astrakhan Oblast	Astrakhan	C108	MH147419	PX146760.1
Astrakhan Oblast	Astrakhan	B3	MH147421	PV980185.1
Astrakhan Oblast	Astrakhan	B3	MH147431	PV980186.1
Astrakhan Oblast	Astrakhan	C1	MH147435	PX146725.1
Perm Krai	Perm	B3	MH148372	PV980199.1
Perm Krai	Perm	B3	MH148376	PV980200.1
Perm Krai	Perm	B7	MH148387	PX121475.1
Perm Krai	Perm	C2	MH148396	PX146729.1
Perm Krai	Perm	B3	MH148410	PV980201.1
Saint Petersburg	Saint Petersburg	B3	MH149423	PV980187.1
Saint Petersburg	Saint Petersburg	B3	MH149429	PV980188.1
Lipetsk Oblast	Lipetsk	C1	MH149917	PX146720.1
Republic of Buryatia	Ulan-Ude	B3	MH166704	PV980189.1
Republic of Buryatia	Ulan-Ude	B3	MH166710	PV980190.1
Republic of Buryatia	Ulan-Ude	B7	MH166714	PX121476.1
Republic of Buryatia	Ulan-Ude	B3	MH166738	PV980194.1
Republic of Buryatia	Ulan-Ude	C89	MH167312	PX146753.1
Republic of Buryatia	Ulan-Ude	B7	MH166717	PX121477.1
Republic of Buryatia	Ulan-Ude	B7	MH166721	PX121478.1
Vologda Oblast	Sheksna	B3	MH166724	PV980191.1
Perm Krai	Perm	C2	MH166725	PX146742.1
Perm Krai	Chaikovsky	B55	MH166726	PV692083.1
Perm Krai	Perm	B3	MH166727	PV980197.1
Perm Krai	Perm	B3	MH166728	PV980192.1
Perm Krai	Perm	B3	MH166729	PV980193.1
Perm Krai	Perm	B3	MH166730	PV980198.1
Krasnodar Krai	Krasnodar	C108	MH166735	PX146759.1
Lipetsk Oblast	Lipetsk	C1	MH166736	PX146722.1
Saint Petersburg	Saint Petersburg	B55	MH209685	PV980162.1
Krasnodar Krai	Krasnodar	C2	MH209686	PX146735.1
Krasnodar Krai	Krasnodar	C1	MH209687	PX146723.1
Krasnodar Krai	Novorossiysk	C2	MH209688	PX146736.1
Krasnodar Krai	Novorossiysk	C89	MH209689	PX146754.1
Krasnodar Krai	Novorossiysk	C5	MH209690	PX146747.1
Krasnodar Krai	Sochi	C2	MH209691	PX146732.1
Krasnodar Krai	Krymsk	B55	MH209692	PV980163.1
Krasnodar Krai	Krasnodar	C89	MH209693	PX146755.1
Krasnodar Krai	Krasnodar	B55	MH209694	PV980164.1
Krasnodar Krai	Krasnodar	C2	MH209696	PX146740.1
Krasnodar Krai	Krasnodar	C89	MH209697	PX146756.1
Krasnodar Krai	Krasnodar	C89	MH209698	PX146752.1
Saint Petersburg	Saint Petersburg	B55	MH209699	PV980175.1
Saint Petersburg	Saint Petersburg	D109	MH209700	PX121480.1
Saint Petersburg	Saint Petersburg	C1	MH209701	PX146721.1
Saint Petersburg	Saint Petersburg	B55	MH209702	PV980177.1
Saint Petersburg	Saint Petersburg	B55	MH209703	PV980165.1
Yamalo-Nenets Autonomous Okrug	Noyabrsk	B3	MH209704	PV980195.1
Yamalo-Nenets Autonomous Okrug	Noyabrsk	C2	MH209705	PX146734.1
Yamalo-Nenets Autonomous Okrug	Noyabrsk	C1	MH209706	PX146724.1
Saint Petersburg	Saint Petersburg	B55	MH209707	PV980166.1
Saint Petersburg	Saint Petersburg	B55	MH209708	PV980167.1
Saint Petersburg	Saint Petersburg	C2	MH209709	PX146731.1
Saint Petersburg	Saint Petersburg	C1	MH209711	PX146727.1
Saint Petersburg	Saint Petersburg	B55	MH209713	PV980168.1
Saint Petersburg	Saint Petersburg	B55	MH209714	PV980169.1
Rostov Oblast	Rostov-on-Don	C5	MH209715	PX146748.1
Saint Petersburg	Saint Petersburg	C1	MH209716	PX146726.1
Khabarovsk Krai	Komsomolsk-on-Amur	C2	MH209717	PX146741.1
Saint Petersburg	Saint Petersburg	B55	MH209718	PV980170.1
Saint Petersburg	Saint Petersburg	C2	MH209719	PX146743.1
Saint Petersburg	Saint Petersburg	B3	MH209721	PV980196.1
Saint Petersburg	Saint Petersburg	B55	MH209722	PV980171.1
Saint Petersburg	Saint Petersburg	B55	MH209724	PV980176.1
Saint Petersburg	Saint Petersburg	B55	MH209725	PV980172.1
Saint Petersburg	Saint Petersburg	B55	MH209726	PV980173.1
Saint Petersburg	Saint Petersburg	B55	MH209727	PV980174.1
Republic of Bashkortostan	Ufa	B55	MH209728	PV980178.1
Saint Petersburg	Saint Petersburg	B55	1092	PV980131.1
Saint Petersburg	Saint Petersburg	B55	1110	PV980132.1
Saint Petersburg	Saint Petersburg	B55	1124	PV980133.1
Saint Petersburg	Saint Petersburg	B55	MH204018	PV980134.1
Saint Petersburg	Saint Petersburg	C2	MH204019	PX146737.1
Saint Petersburg	Saint Petersburg	B3	MH204020	PV980179.1
Saint Petersburg	Saint Petersburg	B55	MH204021	PV980135.1
Republic of Tatarstan	Kazan	B3	MH204023	PV980180.1
Saint Petersburg	Saint Petersburg	B3	MH204024	PV980181.1
Saint Petersburg	Saint Petersburg	C2	MH204025	PX146733.1
Krasnodar Krai	Krasnodar	C2	MH204026	PX146738.1
Saint Petersburg	Saint Petersburg	B55	MH204027	PV980136.1
Saint Petersburg	Saint Petersburg	B55	MH204028	PV980137.1
Saint Petersburg	Saint Petersburg	B55	MH204029	PV980138.1
Astrakhan Oblast	Astrakhan	p5h6f6	MH204031	PX637903.1
Saint Petersburg	Saint Petersburg	B55	MH204032	PV980139.1
Saint Petersburg	Saint Petersburg	B55	MH204033	PV980140.1
Saint Petersburg	Saint Petersburg	B55	MH204034	PV980141.1
Saint Petersburg	Saint Petersburg	B55	MH204035	PV980142.1
Saint Petersburg	Saint Petersburg	p5h57f6	MH204036	PX146749.1
Saint Petersburg	Saint Petersburg	B55	MH204037	PV980143.1
Saint Petersburg	Saint Petersburg	C5	MH204038	PX146744.1
Saint Petersburg	Saint Petersburg	C108	MH204039	PX146728.1
Komi Republic	Syktyvkar	B55	MH204040	PV980144.1
Komi Republic	Syktyvkar	B55	MH204041	PV980145.1
Komi Republic	Syktyvkar	B55	MH204042	PV980146.1
Komi Republic	Syktyvkar	B55	MH204043	PV980147.1
Saint Petersburg	Saint Petersburg	C89	MH204046	PX146750.1
Novosibirsk Oblast	Novosibirsk	C1	MH204047	PX146717.1
Krasnodar Krai	Novorossiysk	B7	MH204049	PX121479.1
Krasnodar Krai	Krasnodar	C2	MH204052	PX146730.1
Saint Petersburg	Saint Petersburg	B55	MH204056	PV980148.1
Saint Petersburg	Saint Petersburg	B3	MH204057	PV980182.1
Saint Petersburg	Saint Petersburg	B55	MH204058	PV980149.1
Saint Petersburg	Saint Petersburg	B55	MH204059	PV980150.1
Saint Petersburg	Saint Petersburg	B55	MH204060	PV980151.1
Saint Petersburg	Saint Petersburg	C108	MH204062	PX146757.1
Saint Petersburg	Saint Petersburg	B55	MH204063	PV980152.1
Saint Petersburg	Saint Petersburg	B55	MH204064	PV980153.1
Saint Petersburg	Saint Petersburg	C1	MH204065	PX146718.1
Saint Petersburg	Saint Petersburg	B55	MH204068	PV980154.1
Saint Petersburg	Saint Petersburg	B55	MH204069	PV980155.1
Saint Petersburg	Saint Petersburg	C108	MH204070	PX146758.1
Saint Petersburg	Saint Petersburg	C1	MH204071	PX146719.1
Saint Petersburg	Saint Petersburg	C2	MH204072	PX146739.1
Pskov Oblast	Pskov	C89	MH204073	PX146751.1
Saint Petersburg	Saint Petersburg	C5	MH204074	PX146745.1
Tambov Oblast	Rasskazovo	B55	MH204075	PV980156.1
Yamalo-Nenets Autonomous Okrug	Noyabrsk	B3	MH167300	PX255345.1
Republic of Buryatia	Ulan-Ude	B7	MH167308	PX255349.1
Republic of Buryatia	Ulan-Ude	B3	MH167309	PX255346.1
Republic of Buryatia	Ulan-Ude	B3	MH167310	PX255347.1
Republic of Buryatia	Ulan-Ude	B7	MH167311	PX255350.1
Republic of Buryatia	Ulan-Ude	C2	MH167315	PX255351.1
Saint Petersburg	Saint Petersburg	B3	MH167320	PX255348.1
Republic of Buryatia	Ulan-Ude	C1	MH166713	PX255352.1
