# Supplementary figures and images for: Nationwide Genomic Surveillance of Human Respiratory Adenoviruses in 2023–2024: Evidence of Extensive Diversity and Recombination in Russia
Source: Viruses. 2026 Jan 21;18(1):136. doi: 10.3390/v18010136 (PMC12846442; doi:10.3390/v18010136)

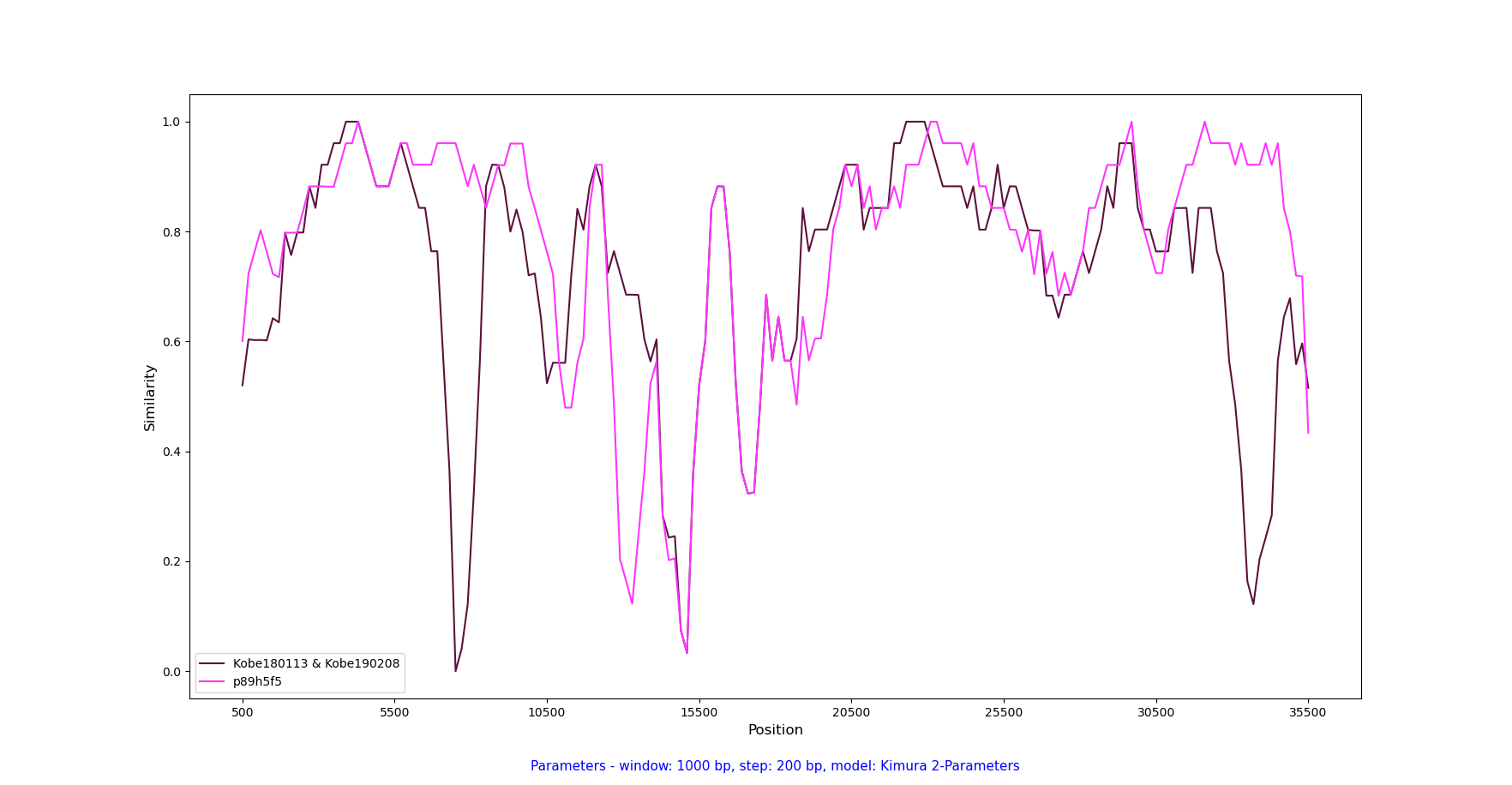

Supplement: Supplementary file 1 [file viruses-18-00136-s001.zip › viruses-4094482-Supplementary_Figure S1.png]

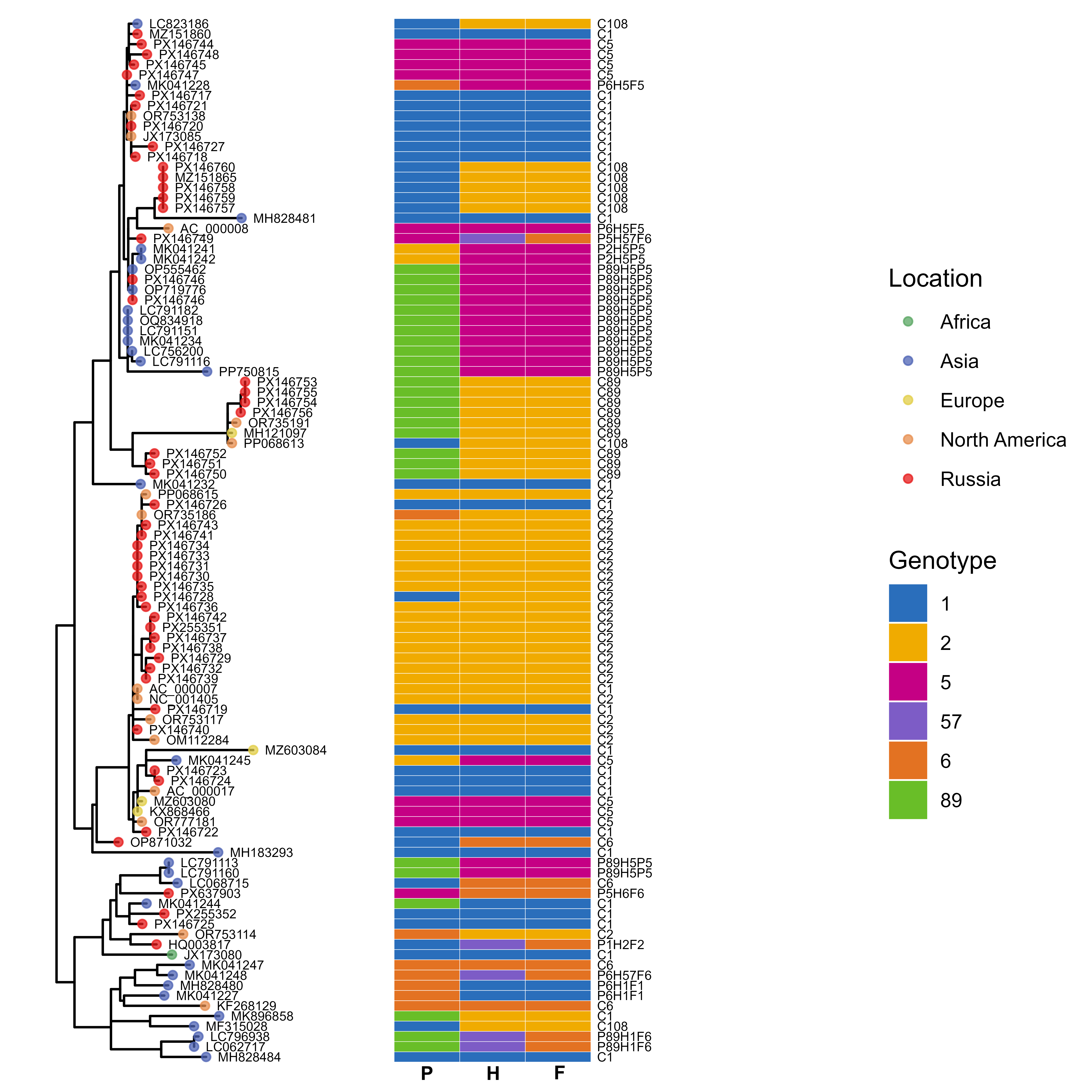

Supplement: Supplementary file 1 [file viruses-18-00136-s001.zip › viruses-4094482-Supplementary_Figure S2.png]
